# “Are You Telling the Truth?” — Testing Individuals’ Ability to Differentiate Between Truth and Deceit in Soccer

**DOI:** 10.3389/fpsyg.2020.01082

**Published:** 2020-05-26

**Authors:** Chris Englert, Geoffrey Schweizer

**Affiliations:** ^1^Institute of Sports Sciences, Department of Sports Psychology, Goethe University Frankfurt, Frankfurt, Germany; ^2^Institute of Education, Department of Educational Psychology, University of Bern, Bern, Switzerland; ^3^Institute of Sports Sciences, Department of Sports Psychology, Heidelberg University, Heidelberg, Germany

**Keywords:** deception, lying, truth, referee, soccer

## Abstract

In the present paper, we tested the ability of individuals to judge correctly whether athletes are lying or telling the truth. For this purpose, we first generated 28 videos as stimulus material: in half of the videos, soccer players were telling the truth, while in the other half, the same soccer players were lying. Next, we tested the validity of these video clips by asking *N* = 65 individuals in a laboratory experiment (Study 1a) and *N* = 52 individuals in an online experiment (Study 1b) to rate the level of veracity of each video clip. Results suggest that participants can distinguish between true and false statements, but only for some clips and not for others, indicating that some players were better at deceiving than others. In Study 2, participants again had to make veracity estimations, but we manipulated the level of information given, as participants (*N* = 145) were randomly assigned to one of three conditions (regular video clips, mute video clips, and only the audio stream of each statement). The results revealed that participants from the mute condition were less accurate in their veracity ratings. The theoretical and practical implications of these findings are discussed.

## Introduction

Antisocial behavior in sports and exercise contexts has been documented in several studies and can be understood as intentional behavior designed to disadvantage other individuals (Kavussanu et al., 2006). Research has focused on, among other topics, the prevalence of antisocial behavior (e.g., Kavussanu, 2006), the use of illegal performance-enhancing drugs (e.g., Momaya et al., 2015), or the reasons why athletes make the decision to display antisocial behavior in the first place (e.g., Kavussanu and Roberts, 2001; Ommundsen et al., 2003). In the present paper, we focus on deception in sports, which involves “making someone believe something that is not true in order to get what you want” (Hsu, 1997), p. 167; for a review, see Güldenpenning et al. (2017). To gain an advantage, athletes are oftentimes motivated to deceive the referee, as deception might change the course of a match, for instance, when a soccer player is asking for a penalty during the last minutes of a match even though there had been no foul (e.g., Traclet et al., 2011; Sabag et al., 2018).

Being able to detect deception is not only relevant during a sporting competition but also in the criminal justice system (e.g., Akehurst et al., 1996) or in educational contexts (e.g., Marksteiner et al., 2013). In fact, most studies on lie detection have been conducted in the context of the criminal justice system, which is not surprising, as it is extremely important to classify a statement correctly in court as being true or false. In general, individuals are not highly accurate when it comes to detecting truths and lies, as a meta-analysis revealed they are only slightly better than chance level (accuracy rate of 54%; e.g., Bond and DePaulo, 2006). Overall, individuals are better at identifying a true statement correctly (accuracy rate of 61%) than at identifying a lie correctly (accuracy rate of 47%). Similar accuracy rates have been reported in the field of sport psychology, for instance, in a study by Renden et al. (2014) in which participants were asked to judge whether tackle situations in soccer matches on television were either fouls or dives. While there are plenty of correlational and qualitative publications on judgment and decision making of sports officials (for an overview see Aragão e Pina et al., 2018), there has been little experimental research on referees’ ability to differentiate correctly between a true statement and an invented one (e.g., Morris and Lewis, 2010). Experimental designs would allow one to draw causal conclusions concerning which factors have a direct influence on referees’ judgment and decision making (e.g., Morris and Lewis, 2010; Sabag et al., 2018).

Therefore, which factors determine whether individuals are capable of estimating accurately the truth of a statement? A meta-analysis by Aamodt and Custer (2006) did not find empirical evidence of a significant effect of gender, age, self-confidence, or certain personality traits (e.g., extraversion) on accuracy rates. Furthermore, expertise does not automatically lead to judgements that are more accurate, meaning that laypersons oftentimes do not differ significantly from experts in their accuracy rates (Aamodt and Custer, 2006; Bond and DePaulo, 2006; del Campo et al., 2018). In sports, results partially suggest that the level of expertise might have an influence on the accuracy rates of judgments (e.g., Renden et al., 2014). However, given the small number of studies on lie detection in sports, future research is needed. Theoretically, the ability to identify correctly both true and false statements hinges on two factors: first, on the presence of cues that differentiate between true and false statements (i.e., valid cues), and second, on individuals’ ability to perceive these cues and to use them in a correct manner (so-called cue usage). This means that individuals must use only valid cues and neglect non-valid cues. Furthermore, they must know how specific cues relate to the probability of a statement being true or false. There are several potential factors which can influence the ability to differentiate between true and false statements [e.g., Need for Cognition (NFC); e.g., Reinhard, 2010]. However, in the present studies, we were primarily interested in (a) developing a valid experimental lie detection research paradigm in sports and (b) investigating individuals’ ability to properly judge critical game situations.

In line with these considerations, Vrij et al. (2006) propose that one potential explanation for the low detection rates seems to be that individuals oftentimes hold inadequate beliefs about valid cues related to deception. For instance, in laypersons and experts, there is a common stereotypic belief that liars have a tendency to avoid eye contact and to display strong nervous body movements (e.g., Bond and DePaulo, 2006). However, DePaulo et al. (2003) did not find any empirical support for these non-verbal and para-verbal cues. Research has repeatedly demonstrated that relying on these invalid cues when making a judgment affects the accuracy rates negatively (e.g., Frank and Ekman, 2004). In general, non-verbal cues, such as the aforementioned ones, are less strongly related to deception than verbal cues (i.e., the content of the respective statement). Several studies identified the following valid verbal cues to deception: lies are not as logically structured as true statements, lies are less plausible, lies do not contain as many relevant details, and lies are more ambivalent than true statements (e.g., DePaulo et al., 2003). Therefore, it seems beneficial to focus on verbal cues instead of non-verbal or para-verbal cues to make an accurate veracity judgment (Forrest et al., 2004).

As previously mentioned, there has been little experimental research in sports-related contexts on antisocial behavior in general and on lie detection in particular. Instead, most studies on antisocial behavior collected data either by interviewing coaches regarding specific behaviors (e.g., Stuart and Ebbeck, 1995) or by asking individuals how they would behave in hypothetical situations (e.g., Kavussanu and Ntoumanis, 2003). One of the only studies that used an experimental design to investigate lie detection in sports was conducted by Morris and Lewis (2010). In their study, they first created five video clips as stimulus material in which they instructed amateur soccer players to exaggerate the effects of a tackle by an opponent in a convincing manner. They videotaped these sequences and asked participants in another study to watch these video clips and to make a judgment regarding the level of exaggeration of the tackled player depicted in the video clip. The results of this study revealed that participants were fairly accurate in estimating the level of exaggeration.

In the present paper, we were not interested in the prevalence of deception or in the reasons why athletes decide to deceive the referee; instead, we focused on the ability of individuals to detect lies. We first created the stimulus material, which consisted of 28 videos in which soccer players were either telling the truth or lying (see also Morris and Lewis, 2010). In Study 1a (laboratory) and Study 1b (online), we tested these video clips by asking participants to rate the veracity of each video (for a similar approach, see Morris and Lewis, 2010). The participants did not see the actual game situation, but only the interview with the respective player which took place after the critical incident. There were two reasons for replicating Study 1a: First, we wanted to make sure that both participants’ ability to discriminate between true and false statements and potential differences between video pairs found in Study 1a reflect systematic differences instead of simply random variation. Second, we wanted to make sure that results do not depend on a laboratory setting, but can also be obtained in an online setting (for a discussion on the replication crisis, see also Klein Richard et al., 2014). In Study 2, we manipulated the type of information presented to the participants, where participants watched the original video clips (i.e., original condition), watched the original video clips without containing any auditory information (i.e., mute condition), or only listened to the audio stream without seeing the actual video clip (i.e., audio condition). In line with previous research, which has shown that non-verbal cues are less reliable than verbal cues (e.g., DePaulo et al., 2003), we assumed that participants from the mute condition would be less accurate in their veracity assessments than participants from the other two conditions. We will explain the stimulus material and the studies in more detail in the following sections. The local ethics committee approved all studies reported in this paper.

## Generation of the Stimulus Material

Fourteen male soccer players (*M*_age_ = 23.36, *SD*_age_ = 4.77) from a club from the sixth highest league in Germany (out of 11 leagues) volunteered to participate to create the stimulus material (for this procedure, see Marksteiner et al., 2015; see also Levine et al., 2011). On average, the players had played soccer for 18 years (*SD* = 3.72), and there were defensive, offensive, and midfield players among them. The study was conducted in single sessions on a regular soccer pitch. We obtained written informed consent from each participant before commencing the study.

**FIGURE 1:**
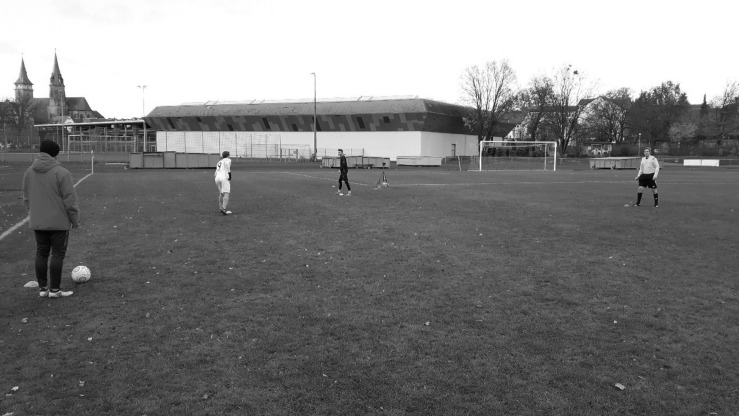
Illustration of the experimental setup for the generation of the stimulus material. The player wearing the jacket is a confederate acting as an attacking player, the player wearing the white jersey is a confederate acting as the teammate of the attacking player, and the player wearing the black jersey is the target player acting as the defender. The referee is standing on the right, observing the scene.

Each participant played the part of a defender twice, leading to two scenarios. In both scenarios, two confederates acted as attacking players from the opposing team (for the setup, see Figure 1). One confederate played a long pass toward the goal line for his teammate (i.e., the second confederate). In one scenario, the defender’s job was to prevent the opposing player from reaching the ball and to let the ball cross the goal line so that the defender’s team would get the goal kick. In the second scenario, however, the defender was instructed to touch the ball slightly before it crossed the goal line, so that the correct decision would actually be a corner kick instead of a goal kick. In both scenarios, after the ball had crossed the line, the referee blew his whistle, requested the defender to come over to a marked position immediately where a video camera was set up, and asked him a series of seven questions (1. Who was the last player to touch the ball? 2. Are you sure? 3. Why is the other player saying something different? 4. Are you sure? 5. Again, who was the last player to touch the ball? 6. Are you sure about that? 7. Why should I believe you?). These questions were developed in cooperation with an official German B-level referee and an A-level soccer coach (both handed out by the German Football Association) to ensure the questions would be as realistic as possible. The defender was instructed to state in both scenarios that the attacker was the last player to touch the ball and that the referee should decide on a goal kick. That way, we generated two videos from each participant: one video in which he was telling the truth and one video in which he was lying (order counterbalanced). The referee was blind to the condition and did not see the actual critical game scene, as he turned away when the tackling happened. To increase participants’ motivation, we offered tickets to a Bundesliga soccer match (first German soccer division) to the one player who was the most convincing. That way, we generated 28 videos in total (14 true statements, 14 lie statements). Each video lasted approximately 28 s (*M* = 27.5, *SD* = 6.27) and contained the same amount of questions asked by the same non-visible referee. Each player’s upper torso, face, and legs could be seen on all video tapes and the sound quality was the same in all video clips.

## Study 1A

The aim of Study 1a was twofold: first, we wanted to investigate the question of whether participants are able to distinguish between true and false statements. Based on existing research, we expected participants to be able to distinguish between true and false statements; however, we expected a small to medium effect size at best (e.g., Bond and DePaulo, 2006). Second, we aimed to determine whether participants’ ability to distinguish between true and false statements differed between video pairs. In other words, we wanted to find out whether some of the players we filmed were better liars than others were. We were interested in determining which video pairs were more ambiguous (i.e., the veracity ratings of the true and untrue statements of the respective target players do not differ significantly) and which video pairs were less ambiguous (i.e., the veracity ratings of the true and untrue statements of the respective target players differ significantly).

### Methods

#### Participants

A total of *N* = 65 university students from a German university participated voluntarily in this study (26 female; *M*_Age_ = 24.66 years, *SD*_Age_ = 4.24). Eight participants had refereeing experience (*M* = 3.88 years, *SD* = 3.87). All participants delivered written informed consent before taking part in the research.

#### Design, Procedure, and Measures

The study was conducted in the university’s laboratory and the videos and all the instructions were administered on a regular computer screen using an online survey software (Unipark). After delivering demographic information (age, sex, mother tongue, and refereeing experience), the 28 videos were displayed in a random order. The participants were informed that in each video clip a player would be asked a series of questions by a professional referee and that they had to rate the veracity of each video clip. All participants were wearing regular stereo headphones and the sound was played at a constant volume. Following each video clip, participants rated the truth of each statement on a continuous scale ranging from 1 (*not at all true*) to 10 (*totally true*; for this procedure, see Marksteiner et al., 2013). Finally, the participants were debriefed and thanked for their participation.

### Results

Overall, participants were able to distinguish between false and true statements. The veracity ratings of false statements (*M* = 5.20, *SD* = 0.78) were lower on average than the veracity ratings of true statements (*M* = 5.81, *SD* = 0.82). A within-subject analysis of variance (ANOVA) indicates that this difference is a significant one, *F*(1,64) = 28.29, *p* < 0.0001, η^2^*_p_* = 0.31. Furthermore, results suggest that participants were indeed able to distinguish between true and false statements for some but not all video pairs.

To test for which video pairs participants were able to distinguish between true and false statements, we conducted within-subject ANOVAs for each video pair. These analyses resulted in seven ambiguous video pairs (i.e., there were no significant differences between the veracity ratings for the true and the deceptive statements) and seven less-ambiguous video pairs (i.e., the veracity ratings for the true and the deceptive statements differed significantly). The detailed results are illustrated in Table 1.

**TABLE 1:** Study 1a: Veracity ratings for the true and the false statements of each video pair.

Video pair number	True statement		False statement			
	*M*	*SD*	*M*	*SD*	*F*	η^2^*_p_*
1	6.08	2.53	5.89	2.61	0.19	0.00
2	5.83	2.52	4.09	2.47	26.27**	0.29
3	6.37	2.36	5.12	2.49	9.34**	0.13
4	4.26	2.32	3.89	2.10	0.87	0.01
5	8.35	1.87	6.46	2.50	22.03**	0.26
6	5.22	2.80	3.55	2.29	17.82**	0.22
7	7.09	2.19	5.62	2.73	14.97**	0.19
8	6.14	2.74	6.60	2.67	1.35	0.02
9	7.06	2.46	6.17	2.88	4.51*	0.07
10	6.26	2.60	5.65	2.70	2.18	0.03
11	3.15	2.39	4.25	2.65	10.66**	0.14
12	6.37	2.44	5.78	2.81	1.83	0.03
13	5.12	2.45	5.71	2.66	2.04	0.03
14	3.97	2.51	4.03	2.22	0.03	0.00

*Note. N = 65. Each video was rated on a continuous scale ranging from 1 (*not at all true*) to 10 (*totally true*). ^∗^*p* < 0.05. ^∗∗^*p* < 0.01.*

### Discussion

The results of Study 1a suggest that, overall, participants are able to distinguish between true and false statements in our stimulus material; however, this ability differs for different video pairs. Furthermore, they revealed that out of the 14 video pairs, seven video pairs were ambiguous, meaning the veracity ratings of the true and deceptive statements of these target players did not differ significantly. On the contrary, for the other seven video pairs, participants were able to differentiate correctly between the true and deceptive statements.

## Study 1B

The primary aim of Study 1b was to test whether the pattern found in Study 1a is robust in a novel study to ensure that both participants’ ability to discriminate between true and false statements and the differences between video pairs found in Study 1a reflects a systematic difference between true and false statements and between video pairs. Furthermore, we wanted to test whether the results depend on a laboratory setting or whether they will also emerge in an online setting (for a discussion on the replication crisis, see also Klein Richard et al., 2014). For this cause, we posted an online link on various social platforms that led participants to an anonymous survey containing all videos and questionnaires (Unipark).

### Methods

#### Participants

In total, *N* = 94 individuals clicked the online link and *n* = 52 individuals finished the study. The individuals who accessed the online survey were informed of the purpose of the study, delivered informed consent, and confirmed that they agreed to participate voluntarily. All the following analyses were conducted only with the participants who finished the study (29 female; *M*_Age_ = 36.54 years, *SD*_Age_ = 15.76). Three participants had refereeing experience (*M* = 3.33 years, *SD* = 4.04).

#### Design, Procedure, and Measures

The design was identical to the design of Study 1a, with the only difference being that Study 1b was conducted online. Participants delivered demographic information and rated the veracity of all 28 videos (Marksteiner et al., 2013). Finally, we thanked the participants for their participation and debriefed them.

### Results

#### Main Analyses

Just as in Study 1a, overall, participants were able to distinguish between false and true statements. The veracity ratings of the false statements (*M* = 5.38, *SD* = 1.21) were lower on average than the veracity ratings of the true statements (*M* = 5.82, *SD* = 1.07). A within-subject ANOVA indicates that this difference is significant, *F*(1,51) = 14.69, *p* < 0.0001, η^2^*_p_* = 0.22.

We again ran within-subject ANOVAs for each video pair to determine which video pairs were ambiguous and which were non-ambiguous (for detailed results, see Table 2). In Study 1b, for seven video pairs, there were no significant differences between the veracity ratings for the true and the deceptive statements (i.e., ambiguous video pairs), while seven other video pairs were non-ambiguous.

**TABLE 2:** Study 1b: Veracity ratings for the true and the false statements of each video pair.

Video pair number	True statement		False statement			
	*M*	*SD*	*M*	*SD*	*F*	η^2^*_p_*
1	5.33	2.80	5.35	2.62	0.01	0.00
2	5.25	2.61	4.96	2.71	0.36	0.01
3	7.02	2.44	5.94	2.49	5.40*	0.10
4	4.58	2.67	5.25	2.88	3.15	0.06
5	7.52	2.41	6.21	2.47	9.88**	0.16
6	6.04	2.61	4.87	2.69	8.74**	0.15
7	6.83	2.47	5.54	2.85	7.64**	0.13
8	5.56	2.73	5.04	2.60	1.55	0.03
9	7.33	2.10	6.60	2.80	3.59*	0.07
10	5.60	3.12	5.67	2.71	0.03	0.00
11	4.06	2.86	5.17	2.69	15.76**	0.24
12	6.17	2.61	5.65	2.66	1.39	0.03
13	5.15	2.52	5.02	2.52	0.11	0.00
14	5.08	2.50	4.08	2.47	9.27**	0.15

*Note. N = 52. Each video was rated on a continuous scale ranging from 1 (*not at all true*) to 10 (*totally true*). ^∗^*p* < 0.05. ^∗∗^*p* < 0.01.*

#### Additional Analyses

As exploratory analyses, given that the basic designs of Studies 1a (laboratory setting) and 1b (online setting) were identical, we merged the data from both studies into a single data sheet and ran additional analyses. A within-subject ANOVA confirmed that overall participants were able to distinguish between false statements (*M* = 5.28, *SD* = 0.99) and true statements (*M* = 5.81, *SD* = 0.94), *F*(1,116) = 42.83, *p* < 0.0001, η^2^*_p_* = 0.27.

Additional within-subject ANOVAs for each video pair were also conducted to determine which video pairs were ambiguous and which were non-ambiguous (for detailed results, see Table 3). Taken together, the analyses confirmed that for seven video pairs there were no significant differences between the veracity ratings for the true and the deceptive statements (i.e., ambiguous video pairs), while for the seven other video pairs there were significant differences (i.e., non-ambiguous video pairs).

**TABLE 3:** Study 1a and b combined: Veracity ratings for the true and the false statements of each video pair.

Video pair number	True statement		False statement			
	*M*	*SD*	*M*	*SD*	*F*	η^2^*_p_*
1	5.74	2.67	5.65	2.61	0.86	0.00
2	5.57	2.56	4.48	2.60	14.13**	0.11
3	6.66	2.41	5.49	2.51	14.74**	0.11
4	4.40	2.48	4.50	2.56	0.11	0.00
5	7.98	2.16	6.35	2.48	31.58**	0.21
6	5.58	2.74	4.14	2.55	26.49**	0.19
7	6.97	2.31	5.58	2.77	22.25**	0.16
8	5.88	2.74	5.91	2.74	0.10	0.00
9	7.18	2.30	6.36	2.84	8.09*	0.07
10	5.97	2.85	5.66	2.70	0.97	0.01
11	3.56	2.63	4.66	2.70	24.45**	0.17
12	6.28	2.51	5.73	2.73	3.25	0.03
13	5.14	2.47	5.40	2.61	0.83	0.01
14	4.46	2.56	4.05	2.33	2.68	0.02

*Note. N = 117. Each video was rated on a continuous scale ranging from 1 (not at all true) to 10 (totally true). ^∗^p < 0.05. ^∗∗^p < 0.01.*

### Discussion

When comparing the results of Study 1a and Study 1b, six video pairs were classified as ambiguous in both studies and six video pairs were classified as non-ambiguous in both studies. In addition, one video pair was classified as ambiguous in Study 1a but not as such in Study 1b, and one video pair was classified as ambiguous in Study 1b but not as such in Study 1a. When merging the data from both studies into a single data file, the results remained stable. The fact that the results of both studies were so similar, even though Study 1a was conducted in a laboratory while Study 1b was conducted online, suggests that our stimulus material is also suited to be applied in future online research.

## Study 2

The aim of Study 2 was to investigate whether the type of information given to the participants influences the accuracy of the veracity ratings. Previous research suggests that verbal behavior (i.e., the content of the statement) is more reliable than non-verbal and para-verbal behavior (e.g., Bond and DePaulo, 2006). This is why we tested the assumption that participants watching mute versions of the 28 video clips would be less accurate in their veracity ratings than participants watching the regular video clips and participants only listening to the verbal statements without actually seeing the video clips.

### Methods

#### Participants

The sample consisted of *N* = 145 students from a Swiss and a German university who volunteered to take part in this study (87 female; *M*_Age_ = 27.03 years, *SD*_Age_ = 7.4); 22 participants had refereeing experience (*M* = 1.85 years, *SD* = 0.36). Before starting the experimental procedure, each participant delivered written informed consent.

#### Design, Procedure, and Measures

The experiment took place in the universities’ laboratories, and the videos, as well as all the instructions, were displayed on a regular computer screen. The general experimental setup was identical to the two previous studies, with the only difference being that we manipulated the type of information given to the participants. Participants were randomly assigned to a condition that included the original video clips (i.e., original condition; *n* = 49), one that included the original video clips without any auditory information (i.e., mute condition; *n* = 47), or one that only included the audio stream of each video clip without any visual information (i.e., audio condition; *n* = 49). The resulting design was a 2 (true vs. false statements) × 3 (original condition vs. mute condition vs. audio condition) mixed design with repeated measurement on the first factor and a between-participants manipulation on the second. In all three conditions, participants were wearing regular stereo headphones and, as in the previous studies, participants rated the truth of each statement on a continuous scale from 1 (*not at all true*) to 10 (*totally true*; Marksteiner et al., 2013). Finally, we thanked the participants for their participation and debriefed them.

### Results

#### Main Analyses

We conducted a 2 (type of statement: true vs. false) × 3 (experimental group: original condition vs. mute condition vs. audio condition) mixed-design ANOVA to test our hypotheses. There was a significant main effect of the type of statement, *F*(1,142) = 10.45, *p* = 0.002, η^2^*_p_* = 0.07. We also found a significant main effect of the experimental group, *F*(2,142) = 9.35, *p* < 0.0001, η^2^*_p_* = 0.12. As expected, there was also a significant interaction between the type of statement (true or false) and the type of experimental group (original condition vs. mute condition vs. audio condition), *F*(2,142) = 6.91, *p* = 0.001, η^2^*_p_* = 0.09. Mean estimations of statements’ veracity suggest that only participants in the original condition and in the audio condition were able to distinguish between true and false statements, but not participants in the mute condition (Table 3). Follow-up *t*-tests suggest that, indeed, participants in the original condition (*t*[48] = 3.14, *p* = 0.003) and in the audio condition (*t*[48] = 3.24, *p* = 0.002) were able to distinguish between true and false statements, whereas participants in the mute condition were not (*t*[46] = -1.42, *p* = 0.16). Importantly, both main effects are qualified by this interaction.

It is an interesting question whether the above-described interaction between the type of statement and the type of experimental group is driven by both the true and false statements or solely by one group of statements. Furthermore, it is an open question whether the finding is driven more strongly by the more ambiguous or the less ambiguous statements. Therefore, we conducted some additional analyses to address these questions. Contrary to the main analysis, these additional analyses were exploratory in nature.

#### Additional Analyses

When looking at the true and false statements separately, data suggest that participants in the original condition and in the audio condition were better at identifying true statements as true than participants in the mute condition. Mean estimates for the true statements (on a scale where high values indicate truth) are higher by almost one standard deviation in the original condition and in the audio condition than in the mute condition (Table 4). As can be expected, a one-way between-group ANOVA delivered a significant main effect of the type of information given on the ratings of the true statements, *F*(2,142) = 10.55, *p* < 0.0001, η^2^*_p_* = 0.17, which can be considered a large effect (Cohen, 1988). There is no such difference for the false statements, *F*(2,142) = 3.03, *p* = 0.051, η^2^*_p_* = 0.04.

**TABLE 4:** Study 3: Mean veracity ratings for the true and false statements, separated by condition (original, mute, audio).

Condition	True statements		False statements	
	*M*	*SD*	*M*	*SD*
Original	5.60	0.79	5.20	0.86
Mute	4.97	0.85	5.10	0.76
Audio	5.89	0.86	5.52	0.99

*N = 145. Each video was rated on a continuous scale ranging from 1 (not at all true) to 10 (totally true).*

Studies 1a and 1b indicated that individuals were unable to differentiate between the true and the deceptive statements in six video pairs, meaning the deceptive statements of six target players were rather difficult to differentiate from their true statements. Therefore, we were also interested in whether the type of information given affected the veracity ratings of these six target players (the high-ambiguous pairs) differently from the veracity ratings of the other eight target players (the low-ambiguous pairs). To investigate this question, we conducted a 2 (type of statement: true vs. false) × 2 (ambiguity of statement: high vs. low) × 3 (experimental group: original condition vs. mute condition vs. audio condition) mixed-design ANOVA with repeated measures on the first and second factors. The crucial triple interaction does not become significant, suggesting that the pattern described above does not differ significantly for the high-ambiguous and low-ambiguous video pairs, *F*(2,142) = 1.28, *p* = 0.282, η^2^*_p_* = 0.018.

### Discussion

Participants in the original condition and in the audio condition were able to distinguish between true and false statements, whereas participants in the mute condition were not. As hypothesized, this pattern suggests that verbal information (i.e., content of statements) is necessary for distinguishing true and false statements, whereas non-verbal behavior alone is not sufficient. Further analyses suggest that the above-described effect was driven primarily by the true statements: participants in the original condition and in the audio condition rated the true statements as being truer, but they did not rate the false statements as being more false. This pattern suggests tentatively that the verbal content of the true statements included cues signaling their truth-value. Alternatively, one might also reason that visual content is not as important in the true statements as in the false statements. We cannot rule out the possibility that a third variable that is confounded with our experimental manipulation might at least partly drive the differences between the experimental conditions. For example, it might be that the experimental manipulations induce different levels of cognitive load (e.g., Paas et al., 2003) or mental fatigue (e.g., Englert, 2019). To the extent that cognitive load as well as mental fatigue influence the ability to distinguish between true and false statements, this would explain the differences between the experimental conditions. Therefore, we suggest that future research utilizes control variables (e.g., a scale assessing cognitive load or mental fatigue) in order to rule out alternative explanations.

## General Discussion

Taken together, results from the three studies suggest that participants are able to distinguish between true and false statements when confronted with our stimulus material. When looking at all 14 video pairs at the same time, the mean difference between the veracity ratings of true and false statements is small, indicating that participants are not very good at discriminating true and false statements, as prior research suggests (e.g., Bond and DePaulo, 2006). Interestingly, however, when looking at all 14 video pairs separately, it becomes apparent that the generally small difference when considering all pairs at the same time is caused by averaging differences of varying sizes: for some video pairs, there are large differences between true and false statements, indicating that participants were able to distinguish well between truth and lies. For other pairs, these differences are smaller, and for some, there are no differences at all, indicating that, on average, participants were unable to distinguish between truths and lies for these pairs. Importantly, this pattern is almost identical in Studies 1A and 1B, suggesting that the differences described above do not merely capture random variation, but constitute systematic differences between videos.

There are two possible (and admittedly speculative) explanations for this pattern of results: first, it might simply be that some video pairs contained valid cues, whereas others did not. Second, it might be that all video pairs contained valid cues, but participants did not use them in some of the videos. For example, participants might not have used some cues because they were not in line with their preconceptions about what constitutes valid cues for lie detection, as suggested by some previous research (e.g., Vrij et al., 2006). The results of Study 2 suggest that the verbal cues were valid, unlike the non-verbal cues. This becomes evident through the observation that the participants in the mute condition were less adept at discriminating between true and false statements. Again, this finding is in line with previous research (e.g., Bond and DePaulo, 2006). We believe that the above described differences between video pairs might be important from theoretical, methodological, and applied perspectives, and thus inspire future research. From a theoretical perspective, future research might shed light onto the reasons underlying differences in discrimination. From a methodological perspective, differences between video pairs emphasize that researchers need to be cautious when averaging single items (e.g., Rose, 2016). From an applied perspective, finally, researchers might want to investigate whether discrimination performance can be improved in general, and for the hard-to-discriminate statements in particular. When there are differences regarding the ratings of the two different videos of one player, it seems obvious to interpret this difference as being caused by the videos’ veracity. However, we consider it important to keep in mind that other differences between the videos might have caused the different ratings.

The current results are in line with the interpretation that participants think that the players were in general truthful and thus rated them as such. It is an open question how our results would look like if participants had assumed that players are generally untruthful. Therefore, we suggest that future research investigates into participants’ presuppositions regarding players’ truthfulness.

The present research has some strengths, but also some limitations. The main strength of the present paper is that we employed an experimental manipulation of lying and telling the truth, which was rather naturalistic at the same time. Obviously, a referee in a real match would never turn away from the action on purpose; however, this was simply our operationalization of a referee not having seen the relevant action. In real matches, it does happen that referees do not see the relevant action, for example, because they were in a position where their line of sight was obstructed. In these situations, referees may well communicate with players. If a scenario like the one from our studies happened in the real world, referees would probably talk to both players involved. In this case, their task would be slightly different from the one employed in our studies: Referees would not necessarily have to judge who is lying and who is saying the truth, but they would have to judge which player they consider to be more trustworthy—even if both of them might appear to be lying (or vice versa). We somewhat simplified this situation for our studies, but the essential task is the same: Judge the veracity of a given statement based on the cues available. Additionally, we consider it to be a strength of the present research that the present findings do not rely on a single study alone but on a set of three studies.

The main limitation of the present research is that, obviously, we cannot make inferences beyond the stimulus material used in our studies. Therefore, we cannot be sure that the findings described above can be generalized to other settings (e.g., other players being filmed, other reasons to lie, or other questions asked). However, we tried to incorporate at least some variation into our stimulus material by filming 14 soccer players and by asking them a set of standardized questions. Additionally, the differences observed between video pairs suggest that we managed to capture at least some variation in answering behavior. Still, future research should replicate the present findings using novel stimulus material. We would also like to mention that our video clips only included male soccer players. Even though previous research from the criminal justice system has revealed that gender does not have a significant effect on veracity judgments (e.g., Bond and DePaulo, 2006), we would recommend a replication of our findings with female soccer players to increase the generalizability.

Future studies might look at a direct comparison between a player who is lying and one who is telling the truth, as suggested above. For this research objective, the scenario from our studies would have to be adapted, so that both players involved can both lie and tell the truth. From a theoretical perspective, this approach might be fruitful not only for research on lie detection in sports, but for research on lie detection generally: In contrast to many other applications of lie detection research, in the situation that we investigated, when one player lies, the other one must be telling the truth. Therefore, this situation allows for investigating into the role of the relative veracity of statements as compared to the role of the absolute one, a comparison that is not possible in many classic lie detection scenarios.

Another important question is which potential factors determine whether individuals have a tendency to rely on valid verbal cues or on stereotypical non-verbal cues. According to Reinhard (2010), NFC is an important factor in this regard. NFC is a personality trait which can be defined as cognitive motivation, meaning the tendency to engage in and enjoy cognitive effort (Cacioppo and Petty, 1982). Previous research has shown that individuals high in NFC primarily base their judgments on valid verbal cues whereas lower levels of NFC are related to a predominant use of non-verbal cues (Reinhard, 2010). Future studies should focus on potential moderators of lie detection performance. One further limitation of the current research is that its relation to real-life situations is not very strong. Thus, we caution against prematurely deriving practical recommendations from this research. Furthermore, we suggest that future research tries to connect to real in-game situations more strongly.

We would also like to acknowledge that our interpretations mostly hinge on comparing the two videos of one pair to each other. That means, we conclude that participants could successfully distinguish lie from truth when the two examples of one video pair differed significantly from each other. However, we did for the most part not take into account the absolute ratings of the respective videos. For example, the ratings of two videos might significantly differ from each other, but both videos are rated in the upper half of the scale, signaling that participants rated both of them being rather true than false, albeit with one of them being rated “truer” than the other. For the goals of our studies, this approach seems to make sense, as we were primarily interested in participants’ ability to distinguish truth from lies, and additionally we were interested into first evidence regarding the cues participants base their ratings on. However, research with different goals will probably have to use different comparisons. For example, researchers interested into the question whether different persons are rated differently regarding their trustworthiness will probably want to look at comparisons between persons, and not between video pairs. Likewise, researchers interested into the question whether certain factors may influence participants’ general willingness to believe that a statement is true or false (i.e., their prior beliefs) will probably want to investigate whether videos’ ratings depend on the variation of the assumed influencing variables. In order to obtain a comprehensive picture of lie detection, researchers would probably have to model veracity ratings as a joint function of (a) participants’ prior beliefs about the likelihood of a statement being true or false (while these beliefs might themselves vary based, for example, on context); (b) person characteristics of the potential liars that have been shown to influence veracity ratings (and which might themselves interact with participants’ characteristics and prior beliefs; (c) cues inherent in the statements themselves (e.g., verbal and non-verbal cues); and (d) contextual factors influencing the ability to detect lies (e.g., contextual load). Such an approach would surely be able to overcome much of the shortcomings of current research on lie-detection and thus it might be able to provide a more comprehensive picture of lie-detection.

Given the still preliminary and not highly applied nature of our research, we are careful to derive any practical recommendations for referees. Our research appears to suggest that referees should not try to deduce lies or truths from non-verbal behavior, but rather should rely on verbal cues. This suggestion is supported by previous research in domains other than refereeing (Vrij et al., 2010), and it is supported by our present data. Given the overall poor ability of humans to discriminate between true and false statements (e.g., Bond and DePaulo, 2006), at least without formal analysis, as employed in legal proceedings, we suggest that basing a decision on a judgment about the veracity of a statement without further information should probably be employed as a last resort only.

## Data Availability Statement

The raw data supporting the conclusions of this article will be made available by the authors, without undue reservation, to any qualified researcher.

## Ethics Statement

Written informed consent was obtained from the individual(s) for the publication of any potentially identifiable images or data included in this article.

## Author Contributions

CE and GS equally contributed to the conceptualization of the studies, review of relevant related work, and writing of the manuscript. Both authors approved the final version of the manuscript and agreed with the order of presentation of the authors.

## Conflict of Interest

The authors declare that the research was conducted in the absence of any commercial or financial relationships that could be construed as a potential conflict of interest.
